# Hypertension in sub-Saharan Africa: a massive and increasing health disaster awaiting solution

**Published:** 2015

**Authors:** Norm RC Campbell, Daniel Lemogoum

**Affiliations:** Departments of Medicine, Physiology and Pharmacology, and Community Health Sciences, O’Brien Institute for Public Health, Libin Cardiovascular Institute, Cumming School of Medicine, University of Calgary, Canada; Douala School of Medicine and Pharmaceutical Sciences, University of Douala, Cameroon, and Erasme Hospital, Free Brussels University, Belgium President of the International Forum for Hypertension Control and Prevention in Africa (IFHA), President of the Cameroon Heart Foundation, and Director of the Cameroon Heart Institute

## Introduction

Increased blood pressure is the leading risk for death globally.^1^ While this is also true in sub-Saharan Africa, there are many hypertension issues that are unique to the region.^2^ A prime and important example is that in most countries in the region, population blood pressure is increasing, while in most countries in the rest of the globe, population blood pressure is decreasing.^3^

Currently hypertension prevalence rates in some sub-Saharan African countries are among the highest in the world, while a few short decades ago, several countries in sub-Saharan Africa had among the lowest blood pressure levels.^2^ Importantly, several sub-Saharan African countries still have hypertension prevalence rates below the global average, providing an important opportunity for prevention. Recently, a needs assessment of hypertension organisations in sub-Saharan Africa also showed important and different needs from hypertension organisations in other regions of the world.^4^,^5^

Dr Seedat has comprehensively outlined relevant issues in his article ‘Why is control of hypertension in sub-Saharan Africa poor?’ (page 193) He concludes by quoting Nelson Mandela ‘We must face the matter squarely … We know that we have it in ourselves as Africans to change all this’.

In my opinion, defining and acknowledging the problem is the most important step, but it is also just the first step in a long journey to improving hypertension prevention and control. That the solution to hypertension in sub-Saharan Africa is within sub-Saharan Africa is another critical observation to start that journey. What are the potential next steps?

## Leadership

Without strong sub-Saharan African champions who will lead and provide direction, little will change. At a recent Pan-African hypertension meeting in Douala, there were several strong hypertension champions with knowledge, vision and skill. Many other strong champions, such as Dr Seedat, reside throughout Africa. These leaders need to work together on the following actions.

## Partnership

There are several important and engaged organisations related to hypertension in sub-Saharan Africa [e.g. International Forum for Hypertension Control and Prevention in Africa, African Heart Network (AHN), Pan-African Society of Hypertension, World Hypertension League African regional office and Pan-Africa Society of Cardiology (PASCAR)]. Much broader partnerships are needed. There is an urgent need to partner with governments and the World Health Organisation (WHO), who have the ability to change policies for prevention and control. The recent worldleading and strong policy to regulate a reduction in salt additives to food in South Africa is a prime example.^6^,^7^

Forming partnerships with primary care, with other non-communicable disease (NCD) groups (e.g. diabetes), and with infectious disease groups and programmes will allow a sharing of limited resources and approaches to people at health risk and this is likely to be more efficient and cost effective. The WHO PENs programme provides a template that could assist in the integration of hypertension control with NCD control.^8^

Civil society organisations are better placed to advocate for societal changes to address fundamental issues such as poverty, corruption and other major social issues that Dr Seedat outlined. Civil society organisations will also have a strong interest in access to medications and basic health needs

Public health and epidemiological organisations are critical to assisting in advocating for improved public health policies that might prevent hypertension. Hypertension leaders need to develop the partnerships required to drive the necessary changes Dr Seedat outlined.

## Strategic planning

A coalition of partner organisations needs to develop and agree to a strategic plan for hypertension prevention and control that is either independent or integrated with NCD prevention and control.^9^ There are several models for hypertension strategies that could be used as examples, and the Expanded Chronic Disease model can be used as a template.^10^-^14^

Recently, many African health organisations supported a fact sheet and call to action on hypertension that could form the basis of the start of a hypertension strategy.^2^ An African hypertension strategy needs broad input and support, as well as prioritisation of actions, based on the importance, feasibility and opportunities for implementing the actions in sub-Saharan Africa.

## Learning from and sharing best practices and experiences

Sub-Saharan Africa is a unique region therefore a sharing of resources, challenges and learnings between countries within the region may be particularly important. External experiences and practices need to be cautiously examined and applied.

As Dr Seedat indicated, expensive technologies and treatment may aggravate problems by using valuable and limited resources that could be used to help many versus a few people. Nevertheless some global experiences, such as the use of task-sharing and treatment algorithms, provide a promise of enhanced outcomes at lower cost, and may be adaptable. Hypertension meetings need to be structured to share best regional experiences in prevention and control.

## Advocacy

Hypertension organisations in general are not in a position to make decisions that would influence hypertension prevention and control. Therefore advocacy plays a critical role. Usually advocacy is more effective aligned with partners who agree to common goals (e.g. the need for a reliable, affordable supply of medications). Advocacy needs to be a major part of the implementation of a hypertension strategy. The recent United Nations meeting where most countries signed on to improve control of hypertension by 25% by 2025, and to increase access to essential medication and technologies, represents an important advocacy opportunity.^15^

## Conclusion

The increasing prevalence of hypertension and poor control rates in Africa represent a complex problem. A well-organised, strategic approach with a broad partnership is the best opportunity for improvement. As Dr Seedat indicates, complex societal issues and especially poverty and lack of resources make the task daunting, but emphasise the importance of partnerships, strategic approaches and advocacy.

While the solution to hypertension prevention and control resides within Africa, global hypertension organisations stand supportive to provide what expertise and knowledge we have. The World Hypertension League (WHL), while resource poor, is very interested in working with sub-Saharan African health organisations. Dr Lemogoum, a board member of the WHL, has recently opened a regional office of the WHL in Cameroon. At the end of 2014, the WHL supported academic training sessions on blood pressure screening, strategic planning for reducing dietary salt, and strategic planning to control hypertension at the 7th African Scientific Meeting on Hypertension and Cardiovascular Protection, sponsored by the International Forum for Hypertension Control and Prevention in Africa in Douala, Cameroon.

In 2015, with the assistance of African health organisations and experts, the WHL led the development of a fact sheet and call to action, an infogram and a region-specific needs assessment. The International Society of Hypertension supported and co-sponsored many of these resources. The WHL is strongly encouraging national organisations to develop fact sheets and calls to action and has written a manuscript instructing how to do so.^16^ Furthermore, the WHL has developed a template for strategic planning.^10^ The WHL looks forward to working with regional sub-Saharan African organisations to prevent and control hypertension.
